# Go with the Flow—Trophoblasts in Flow Culture

**DOI:** 10.3390/ijms21134666

**Published:** 2020-06-30

**Authors:** Beatrice A. Brugger, Jacqueline Guettler, Martin Gauster

**Affiliations:** Division of Cell Biology, Histology and Embryology, Gottfried Schatz Research Center, Medical University of Graz, Neue Stiftingtalstraße 6, F/03/38, 8010 Graz, Austria; beatrice.brugger@medunigraz.at (B.A.B.); jacqueline.serbin@medunigraz.at (J.G.)

**Keywords:** pregnancy, placenta, development, trophoblast, flow culture

## Abstract

With establishment of uteroplacental blood flow, the perfused fetal chorionic tissue has to deal with fluid shear stress that is produced by hemodynamic forces across different trophoblast subtypes. Amongst many other cell types, trophoblasts are able to sense fluid shear stress through mechanotransduction. Failure in the adaption of trophoblasts to fluid shear stress is suggested to contribute to pregnancy disorders. Thus, in the past twenty years, a significant body of work has been devoted to human- and animal-derived trophoblast culture under microfluidic conditions, using a rather broad range of different fluid shear stress values as well as various different flow systems, ranging from commercially 2D to customized 3D flow culture systems. The great variations in the experimental setup reflect the general heterogeneity in blood flow through different segments of the uteroplacental circulation. While fluid shear stress is moderate in invaded uterine spiral arteries, it drastically declines after entrance of the maternal blood into the wide cavity of the intervillous space. Here, we provide an overview of the increasing body of evidence that substantiates an important influence of maternal blood flow on several aspects of trophoblast physiology, including cellular turnover and differentiation, trophoblast metabolism, as well as endocrine activity, and motility. Future trends in trophoblast flow culture will incorporate the physiological low oxygen conditions in human placental tissue and pulsatile blood flow in the experimental setup. Investigation of trophoblast mechanotransduction and development of mechanosome modulators will be another intriguing future direction.

## 1. Hemochorial Placentation and Fluid Shear Stress

Human gestation involves so-called hemochorial placentation, which means that maternal blood is in direct contact with the fetal part of the placenta—the chorion frondosum, consisting of placental chorionic villi. However, before perfusion of the placenta with maternal blood, and thus hemochorial placentation, is fully established, a number of fundamental processes occur. Three to four days after fertilization, the morula stage is defined by the occurrence of a totipotent cell mass consisting of approximately sixteen cells. Still in the fallopian tube, but only one day later, cells of the morula differentiate into an inner and an outer cell mass, referred to as the embryoblast and the trophoblast, respectively. Entrance of the blastocyst into the uterine cavity is followed by apposition and adhesion of the blastocyst with its embryonic pole to the endometrial epithelium, enabling implantation of the embryo into the maternal endometrium and subsequent initiation of placentation. As soon as adhesion of the blastocyst is established, trophoblasts located at the embryonic pole (now equivalent with the “implantation pole”) start to fuse to form a multinucleated syncytium, referred to as the syncytiotrophoblast. At that very early stage of embryo implantation, the syncytiotrophoblast is the cell type that enables penetration of the endometrial epithelium and the underlying stroma, which now is referred to as decidua basalis. Once the blastocyst has completely infiltrated the decidual stroma, the syncytiotrophoblast rapidly increases in size by continuing proliferation and fusion of underlying mononucleated cytotrophoblasts. Shortly thereafter, primary placental villi, composed of a cytotrophoblast core and an overlying syncytiotrophoblast layer arise. At the distal regions of these primary villi, cytotrophoblasts breach the syncytiotrophoblast, differentiate into an invasive phenotype, and invade as so-called extravillous trophoblasts the decidual interstitium up to the first third of the myometrium. During migration, extravillous trophoblast subpopulations encounter and invade several luminal structures, including uterine spiral arteries, and veins (endovascular trophoblasts), glands (endoglandular trophoblasts), and to a minor extent uterine lymphatic vessels (endolymphatic trophoblasts) [1,2,3]. This way, arteries, veins, and glands are connected to the intervillous space to guarantee successful placentation. However, before uteroplacental blood flow is completely established, extravillous trophoblasts accumulate and form cellular plugs that largely obstruct maternal arterial blood flow into the intervillous space until the end of the first trimester of pregnancy. At gestational week six to seven, these trophoblast plugs appear loosely cohesive with clear capillary-sized channels, enabling constant microvascular flux into the intervillous space [4]. Thus, a distinct functional relevance can be attributed to trophoblast invasion into spiral arteries, which results in the remarkable remodeling of vessels, including depletion of smooth muscle cells and loss of the elastic lamina in their walls. The consequence thereof is that opening of spiral arteries into the intervillous space dilate and resemble flaccid conduits, enabling reduction of the velocity of incoming maternal blood and thereby preventing damage to delicate villous trees [5]. 

**Figure 1:**
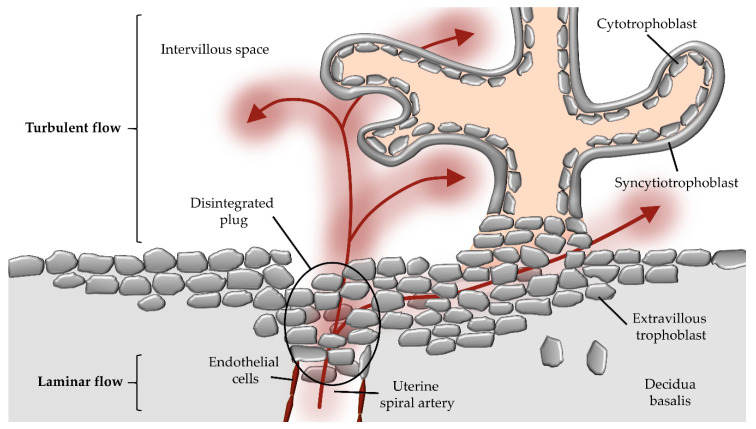
Potential route of maternal blood flow into the intervillous space during first trimester. During early placental development, maternal blood flow in maternal uterine spiral arteries is obstructed by extravillous trophoblast plugs. However, parts of maternal blood (red arrows) can pass through narrow intertrophoblastic gaps when trophoblast plugs begin to dissolve during first trimester of gestation. The laminar blood flow from the maternal spiral artery changes to a turbulent flow upon entrance into the intervillous space.

With hemochorial placentation, and thus establishment of maternal blood flow, the perfused fetal chorionic tissue has to deal with fluid shear stress, which is produced by plasma and hemodynamic forces across uteroplacental endothelial cells and trophoblast subtypes throughout gestation. Based on a simplified model, fluid shear stress in blood vessels is quantified by the dimension of the inner diameter of the vessel, velocity of flow, and dynamic viscosity, resulting in the force per unit area (dyn/cm² = 0.1 Pa) [6]. However, the pulsatile maternal blood flow, the dynamic viscosity of maternal blood, and the micro-anatomical architecture of uterine blood vessels and structure of placental villous trees complicate appraisal of the in vivo fluid shear stress in human utero-placental circulation [7]. Thus, fluidic flow at the uteroplacental interface can be laminar and/or turbulent, resulting in variations in intraluminal forces (Figure 1).

Cells are able to sense fluid shear stress through mechanotransduction, which activates multiple downstream signaling pathways. In addition to endothelial cells, mechanosensing has recently been reported for many other cell types, including trophoblast subtypes that are exposed to fluidic flow. A variety of proteins, receptors, and transmembrane channels are suggested to act as mechansosensors [7,8]. Besides G-protein coupled receptors, integrins, and ion channels, the cytoskeleton of the cell may be involved in mechanosensing. Accordingly, fluidic flow may deform the cellular surface, leading to transduction of forces to cytoskeletal filaments through membrane-spanning proteins, focal adhesion proteins, and glycocalyx components, such as heparin sulfate, chondroitin sulfate, and hyaluronic acid moieties. This interconnection of cellular mechanosensors and cytoskeletal components has led to the concept of a cellular mechanosome complex [8].

During human reproduction, even the preimplantation embryo, including the morula and later on the early blastocyst, is considered to be subject to fluidic flow forces generated by peristalsis of the fallopian tube. However, at this early phase of conception, the zona pellucida — a thick glycoprotein layer, which surrounds the oocyte to allow only species-specific fertilization and persists up to the early blastocyst stage — is suggested to protect the early embryo from harmful mechanical forces [9]. Only with the onset of intervillous perfusion, hemodynamic forces affect the phenotype and physiology of extravillous trophoblasts in invaded uteroplacental spiral arteries as well as villous trophoblasts covering placental villi. Failure of adaptions to this fluid shear stress is suggested to contribute to pregnancy disorders, including fetal growth restriction, which manifests upon impaired spiral artery remodeling, high vascular resistance, and placental hypoperfusion [7].

## 2. Flow Culture Approaches in Trophoblast Research

While in the early twentieth century basic principles for plant and animal cell cultures in vitro were developed (see comprehensive overview by Magdalena Jedrzejczak-Silicka http://dx.doi.org/10.5772/66905), first reports about continuous-flow culture of mammalian cells date back to the 1950s, which describe cells grown in suspension [10]. Later on, in the late 1970s, flow culture of periodontium explants from adult mouse, even under different pO2 ranges, has been reported [11]. During the past twenty years, a remarkable body of work has been devoted to adherent cell culture in microfluidic channels, which usually are designed in micron length-scales and are developed to generate well-defined microenvironments with various patterns of fluidic flow (pulsatile, steady, or oscillatory) [12]. By using such fluidic cell culture systems, a wide panel of different cell types, including stem cells, fibroblasts, endothelial cells, osteoblasts, smooth muscle cells, hepatocytes, cancer cells, and neuronal cells have been analyzed [13]. In addition, human- and animal-derived trophoblasts were subjected to fluidic flow culture, using rather diverse experimental setups (Table 1 and Table 2). While used trophoblast-derived cell lines include the choriocarcinoma cell lines JAR [14], JEG-3 [15], and BeWo [16,17], as well as the Simian Virus 40 (SV40)-transformed trophoblast cell lines HTR-8/SVneo and SGHPL-4 [14,18], primary trophoblasts have been used after isolation from first trimester [19,20] and term placenta [21,22,23]. Besides human trophoblasts, primary macaque trophoblasts [23,24] have been used due to anatomical similarities between human and macaque placental tissues. Moreover, rabbit trophoblast progenitors derived from blastocyst obtained four days post coitum from New Zealand White female rabbits extend the long list of trophoblast cells used for fluidic flow experiments [17].

According to literature, trophoblasts have been subjected to a rather broad range of different flow rates and shear stress values, ranging from the µl- to ml/min scale and 0.001-30 dyn/cm², respectively. These variations in the experimental setup may reflect the general heterogeneity in blood flow through different vessel segments, with a mean shear stress of approximately 7.5 dyn/cm² in large veins, about 15 dyn/cm² in large arteries, and 30 dyn/cm² within venules and arterioles [22]. For the uteroplacental circulation, maternal blood flow through invaded uterine spiral arteries has been suggested to be 1–10 dyn/cm², whereas the flow rate is drastically reduced to 0.001– 0.1 dyn/cm² after entrance into the wide cavity of the intervillous space, and is assumed to peak at roughly 2 dyn/cm² in some areas [16,25,26]. The shear stress exerted on different areas of the syncytiotrophoblast surface may vary, as the intervillous space is a highly asymmetric open space and chorionic villi show a very complex structure [25]. Therefore, different regions within the intervillous space (proximal or distal to the spiral arterial opening), and even different parts of a villous tree (free-floating or anchoring villi) may be faced with different dimensions of shear stress. Since fluid shear stress depends on the dimension and architecture of the vessel, design of the used cell culture dish is a critical aspect for the experimental setup. Depending on the addressed research questions, authors used various different systems ranging from commercially available 2D flow chambers to borosilicate glass capillary tubes and customized 3D micro-scale plastic ware solutions. Beside commercially and customized culture devices, the use of dextran microcarrier beads in combination with fluid shear stress produced by a rotating wall vessel bioreactor has been described to achieve a 3D flow culture model [27]. Moreover, different flow protocols, including open systems and closed circuits, different protein surface coatings (e.g., collagen type I [16,17,20,22,23,24], and fibronectin [14,18,28]) and different incubation times from only minutes [16] up to 96 h [17] and even 21 days [27] have been described.

Thus, study questions, such as to how fluidic flow and shear stress influences different aspects of trophoblast physiology (e.g., differentiation and fusion (Table 1) and migration (Table 2)), should be addressed with a most appropriate setting of the flow system.

## 3. The Influence of Fluid Shear Stress on Trophoblast Turnover and Differentiation

Human placenta development relies on a tightly controlled villous trophoblast turnover, which involves proliferation, differentiation, and fusion of mononucleated cytotrophoblasts with the overlying syncytiotrophoblast [14]. This process guarantees that the syncytiotrophoblast is continuously supplied with cytoplasm and organelles derived from the fusing cytotrophoblasts. Acquisition of newly incorporated cell components is balanced by a concomitant release of apoptotic material as syncytial knots from the syncytiotrophoblast surface into the maternal circulation [30]. Effects of flow and fluid shear stress on trophoblast turnover, in particular on cell differentiation, are manifested by changed cell morphology. Fluid shear stress is suggested to activate signaling pathways involved in trophoblast differentiation and syncytialization by increasing levels of intracellular cyclic adenosine monophosphate (cAMP) and activated cAMP response element-binding protein (CREB) (Table 1, [21]). Activation of cAMP signaling induces upregulation of transcription factor glial cell missing 1 (GCM1) and its downstream targets syncytin-1 (ERVW-1) and syncytin-2 (ERVFRD-1), both of which well-accepted fusogens involved in trophoblast syncytialization [30]. Concurrent to syncytialization is the loss of epithelial junctional- and cytoskeletal proteins, such as E-cadherin, desmoplakin, and α-fodrin [31]. While previous flow culture experiments clearly showed a network of continuous and well-defined junctional complexes in unstimulated trophoblasts after three days perfusion (Table 1, [29]), knowledge on cytoskeleton remodeling upon syncytialization under flow rates is rather limited.

Increased wall shear stress is suggested to act at the villous surface in the inflow regions of the intervillous space, where high wall shear stress could damage the villous trophoblast or at least affect its cellular turnover. This disturbance may be reflected in enhanced trophoblast shedding and elevated levels of free fetal DNA in the maternal circulation [32]. Paradoxically, increasing shear stress has been suggested to have a protective effect against induced apoptotic death in trophoblast cell lines [18]. This has been shown in mononucleated (i.e., undifferentiated) JAR and SGHPL-4 cells, which underwent less apoptosis when cultured under 3 dyn/cm² than those in 0.5 dyn/cm² cultures [18]. Moreover, trophoblasts have been shown to have a survival advantage over endothelial cells. Trophoblasts cultured on human umbilical vein endothelial cells (HUVECs) monolayers at 0.5 or 3 dyn/cm² significantly induced apoptosis in directly adjacent HUVECs, by Fas/Fas-ligand mediated mechanisms [18]. 

In addition to apoptosis, fluid shear stress is suggested to influence trophoblast fusion. Previous studies with BeWo cells and rabbit trophoblastic stem cells (rTSCs) showed increased cell fusion under fluid shear stress. This has been demonstrated for rabbit trophoblastic stem cells at flow rates of 0.1 ml/min, 0.2 ml/min, and 0.5 ml/min, respectively. The authors of the study described that fusion of rabbit trophoblasts occurred between more than two cells, while in the case of BeWo it was mainly a fusion of only two cells (Table 1, [17]). However, at this point it should be noted that BeWo cells occasionally contain two nuclei, and that intercellular fusion must be distinguished from endoreduplication [33], which represents replication of the nuclear genome in the absence of mitosis, and therefore results in an elevated nuclear gene content and polyploidy. However, besides syncytialization, other signs of trophoblast differentiation have been observed when cells were cultured under fluidic flow. According to Miura et al., BeWo cells and human villous trophoblasts react on fluid shear stress by abundant formation of microvilli, which vary in lengths depending on the flow rate (Table 1, [16]). At the center of the chamber, where the shear stress was low (0.001 dyn/cm²), microvilli were long (>2 µm); whereas they were shortened (<2 µm) in the area at the inlet or outlet of the chamber with high shear stress (0.1 dyn/cm²). In agreement with this observation, ezrin—a member of the ezrin-radixin-moesin (ERM) family, which plays a major role in formation and/or maintenance of actin-based cell surface structures—was predominantly detected at the apical membrane of the cells.

The observation of fluid shear stress-induced microvilli formation has recently been confirmed in rabbit trophoblast stems cells, which were cultured on a collagen gel in the presence of flow (Table 1, [17]). Subsequent transcriptome analysis of the rabbit trophoblasts, showed enrichment in pathways regulating actin cytoskeleton and sphingolipid metabolism, which has been suggested to account for the increased formation of microvilli during differentiation [17]. 

## 4. The Influence of Fluid Shear Stress on Trophoblast Metabolism

While the transfer of gases and some other solutes occurs by flow limited diffusion, nutrients, and waste products have to be actively transported across the placental barrier. The extent of nutrient transfer and, hence, of fetal supply is determined by many factors, including placental morphology as well as uteroplacental and fetoplacental blood flow [34]. Perazzolo et al. suggested that the relationship between maternal blood flow and villous structure affects the efficiency of placental uptake and transfer, and moreover, that flow rate may be the major determinant of it [35]. However, besides uteroplacental blood flow, barrier thickness, and concentration gradients, factors such as transporter expression and metabolism of the villous trophoblast influence the dynamics of placental transfer. In fact, a growing body of evidence suggests substantial differences in metabolism in cells cultured under flow, when compared to static conditions. Accordingly, fluidic flow increased the accumulation and size of lipid droplets in rabbit trophoblasts [17], suggesting that morphological differentiation was accompanied by metabolic changes. Consistent with morphological differentiation of rabbit trophoblasts and their increased lipid droplet accumulation, a number of genes of the peroxisome proliferator-activated receptor (PPAR) signaling pathway were upregulated in response to shear stress. Amongst these genes, perilipin 2 (PLIN2), cytochrome P450 1B1 (CYP1B1), and angiopoietin-like 4 (ANGPTL4) are reported to be involved in lipid metabolism, transport and storage (Table 1, [17]), suggesting enhanced metabolic turnover in trophoblasts cultured under flow. In addition to its importance for the formation of lipid droplets, PLIN2 is necessary for trophoblast viability when exposed to fatty acids. This has been shown by overexpression of PLIN2 in human primary term trophoblasts that were exposed to a mixture of linoleic acid and oleic acid [36].

Along with lipid metabolism, trophoblastic glucose uptake is affected by flow, as shown by significantly increased uptake of the fluorescent glucose analog 2-[N-(7-nitrobenz-2-oxa-1,3-diazol-4-yl)amino]-2-deoxy-D-glucose (2-NBDG) into BeWo cells exposed to fluid shear stress. The increased glucose uptake could be explained by slightly increased mRNA expression of the glucose transporter type 1, GLUT1 (encoded by *SLC2A1*), which predominantly localized to the apical membrane of cells and cell–cell contact regions after overnight exposure to fluid shear stress [16].

## 5. The Influence of Fluid Shear Stress on Trophoblast Endocrine Activity 

Soon after implantation, the developing placenta starts producing hormones to adapt the maternal physiology to the progressing pregnancy. The highly differentiated syncytiotrophoblast is the predominant cell type involved in placental hormone synthesis, and thus it is obvious that maternal blood flow may not only affect trophoblast differentiation and metabolism, but also its endocrine activity. Recent studies indicate that fluidic flow induces both, syncytialization and production of the classical pregnancy hormone human chorionic gonadotropin (hCG) in BeWo cells [17]. Moreover, laminar and continuous fluid shear stress of 1 dyn/cm² promotes placental growth factor (PGF) upregulation in primary human term trophoblasts, which underwent spontaneous differentiation and fusion during a 48 h pre-culture under static conditions (Table 1, [21]). At the same time, secretion of soluble fms-like tyrosine kinase-1 (sFlt-1)—suggested as a biomarker to predict the risk of developing preeclampsia [37]—was only slightly, but not significantly increased by fluid shear stress [21].

Moreover, fluid shear stress is suggested to affect the intracellular availability of cortisol, a glucocorticoid that is regulated by 11β-hydroxysteroid dehydrogenase enzymes (11β-HSDs). In human trophoblast cells, this has been shown for 11β-HSD2 (encoded by *HSD11B2*), an NAD^+^-dependent enzyme that oxidizes cortisol to the inactive metabolite cortisone. In JEG-3 cells, a unidirectional flow environment with varying fluid shear stress equal to a maximum of 5 dyn/cm^2^ reduced 11β-HSD2 mRNA expression and activity, which was reversed to basal levels by discontinuation of the shear stress [15]. Conversion of cortisol to cortisone by 11β-HSD2 is suggested to protect cells from the growth-inhibiting and/or pro-apoptotic effects of cortisol, particularly during embryonic development. Thus, fluid shear stress could be one of the underlying causes of enhanced cortisol levels and reduced 11β-HSD2 activity in fetal growth retardation [38].

## 6. The Influence of Fluid Shear Stress on Trophoblast Motility 

Early after blastocyst implantation and initial development of primary placental villi, extravillous trophoblasts detach from villi and start to invade into the decidual stroma. Once these extravillous trophoblasts have eroded uterine spiral arteries (now referred to as endovascular trophoblasts [1]), they are suggested to migrate along the luminal surfaces of the vessels and remodel them by interdigitating between the endothelial cells [14]. Thereby, endothelial cells are increasingly replaced and most of the musculoelastic tissue in the vessel walls dissolve, resulting in low-resistance vessels to guarantee constant and maximal uteroplacental blood flow at the transition from the first to second trimester of pregnancy. Hence it is more than likely that fluidic flow not only influences physiology of the villous trophoblast, but also that of endovascular trophoblasts located in invaded spiral arteries. Importantly, with the erosion of the uterine vessel walls and the phenotypic switch from interstitial extravillous trophoblasts into endovascular trophoblasts, cells are exposed to a much higher fluid shear stress than the subpopulation of villous trophoblasts that are faced with rather low shear stress in the intervillous space. In the context of spiral artery remodeling, a vast majority of studies used co-cultures of extravillous trophoblast cell lines (Table 2) with endothelial cells, such as HUVEC [14,18,20,28] and human uterine microvascular endothelial cells (UtMVEC) [23,24]. On the basis of such co-cultures, as well as trophoblast monocultures, a growing body of evidence suggests that in particular motility of endovascular trophoblasts is controlled by flow. However, it should be noted here that used extravillous trophoblast cell lines (Table 2) seem to be the right choice for such studies, although it remains to be discussed to what extent they resemble the endovascular phenotype.

Initial experiments with early gestation macaque trophoblast cells exposed to flow, showed that cells exhibit clear migration in the direction of flow as well as shape changes that involve extension and retraction of filopodia at its leading edge (Table 2, [22]). In doing so, macaque trophoblast migration velocity and movements increased with the magnitude of the applied shear stress (from 7.5 and 15 up to 30 dyn/cm²). These observations have been confirmed by others, showing that migration of the human first trimester trophoblasts occur generally in the direction of flow (up to 30 dyn/cm²), with only a few cells migrating against the flow stream [19]. The enhanced motility under fluid shear stress seems to be accompanied by increased expression of integrin β1, which mediates adhesion of human and macaque trophoblast cells to endothelial cells in vitro [39]. This has been shown in previous migration experiments, suggesting that factors expressed on the surface of uterine endothelial cells and factors released by the endothelium regulate trophoblast migration under flow [23]. While the extent of migration against flow at higher and more physiological shear stress levels (15 and 30 dyn/cm²) decreased significantly for macaque trophoblasts alone, migration against flow remained virtually unchanged for trophoblasts co-cultured on human uterine microvascular endothelial cells. Hence, migration behavior of trophoblasts cultured under flow clearly depends on the substrate they are seeded.

When SGHPL-4 trophoblasts were cultured on the extracellular matrix glycoprotein fibronectin or a layer of endothelial cells, they did not undergo directional migration in 0.5 and 2 dyn/cm² cultures. However, under conditions of 4 and 6 dyn/cm², trophoblasts migrated with the direction of flow [14]. In contrast, another study suggests that the average migration velocity of human first trimester trophoblasts cultured on a type I rat collagen-coated surface increased almost linearly with increasing shear stress (from 7.5 up to 30 dyn/cm²) (Table 2, [20]), whereas migration velocity remained almost unchanged at all levels of shear stress when cells were cultured on endothelial cells. Similar behavior was observed for the displacement of trophoblasts by flow. The absolute x-direction displacement increased with increasing shear stress, when trophoblasts were cultured on a collagen-coated surface. However, when cultured on top of an endothelial cell monolayer, first trimester trophoblasts showed a strong ability to withstand displacement by flow. Subsequent flow experiments with neutralizing antibodies suggested that integrin β1 regulated adhesion of trophoblasts to endothelial cells under shear stress [20]. However, another study using SGHPL-4 cells suggested that fluid shear stress did not affect expression of the adhesion molecules E-selectin and integrins α4, β1, and αVβ3 [14]. Different cell types and particularly varying fluid shear stress values exposed to cells may explain discrepancies in motility and expression of adhesion molecules. Accordingly, James et al. recently suggested a scenario that extravillous trophoblasts in early first trimester do not undergo directional migration under very low shear stress, whereas later on, as shear stress increases with complete dissolution of trophoblast plugs, the number of trophoblasts are stimulated to migrate in the direction of flow [14].

## 7. Outlook—Future Directions 

**Figure 2:**
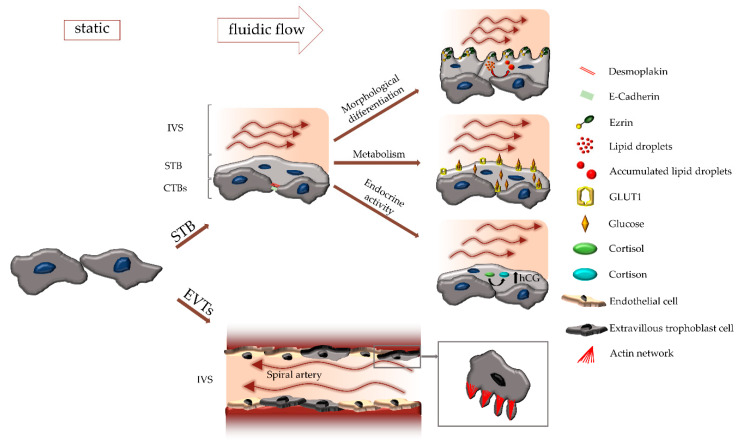
Influence of fluidic flow on different trophoblast subtypes. Fluidic flow regulates differentiation and physiology of both, the syncytiotrophoblast (STB) and extravillous trophoblasts (EVTs). During syncytialization, cell-cell contact proteins, such as desmoplakin and E-cadherin are downregulated, and the structural protein ezrin relocalizes from the basal to the apical side. Formation and appearance of microvilli on the apical surface as well as accumulation of lipid droplets in the STB are influenced by fluidic flow. Moreover, fluidic flow affects expression and localization of GLUT1, secretion of hCG as well as conversion of cortisol to cortisone. In the EVT subpopulation, formation of filopodia, and hence migratory behavior is regulated by fluidic flow as well.

In recent years a number of excellent studies have yielded in an increasing body of valuable knowledge, substantiating an important influence of maternal blood flow on the (patho-)physiology of different trophoblast subtypes. The application of different flow culture systems, including customized micro-scale 3D flow chambers, has revealed substantial differences of trophoblasts cultured under flow, when compared to cells under static conditions (Figure 2).

Some important key parameters should be considered when setting up a trophoblast flow system *:

(1) Reservoir
  - a single reservoir intercalated in a circulating flow system
  - a single reservoir with fresh medium and a tank for consumed medium
  - additional reservoirs with buffer between pump and chamber to damp flow
(2) Pump
  - Peristaltic pump for circulating flow loop systems
  - Syringe pump or electropneumatic pump for one-time inlet-to-outlet flow systems
  - Rotating wall vessel bioreactor (rotation is responsible for distribution of medium)
(3) Chamber
  - Commercial flow chamber (laminar or turbulent)
  - Customized Chamber (laminar or turbulent)
  - Chambers created with 3D printers (suitable for sterilization)
(4) Tubings
  - Size is related to flow rate
(5) Culture medium
  - Serum-free (lower viscosity)
  - With serum (higher viscosity)
(6) Microscope
  - continuous microscopic recording of cultivated cells

* after setup, flow systems should be calibrated prior to each experiment

Besides fluidic flow, a low oxygen environment is another crucial key regulator of trophoblast- and placenta development. In the first trimester of human gestation, prior to fully established uteroplacental blood flow, the mean pO2 in the developing placenta is suggested to be less than 20mmHg. At the beginning of the second trimester, when extravillous trophoblast plugs almost completely disintegrate, pO2 rises to values above 50 mmHg and stays at about this level until delivery [40,41]. Thus, future trends in trophoblast flow culture should incorporate appropriate oxygen conditions in the experimental setup. Moreover, a physiological pulsatile blood flow, leading to temporal and spatial variations of the wall shear stress can be considered to result in different phenotypes and functions of cells. While studies in the field of vascular tissue engineering increasingly acknowledge the importance of temporal and spatial variations of the wall shear stress on endothelial cells, this aspect has not yet been considered for trophoblast culture so far.

Since composition of the used culture medium and serum supplementation will greatly influence the trophoblastic phenotype, care should be taken in medium selection to meet specific requirements of different trophoblast subtypes. Recent studies suggest that extravillous trophoblasts penetrate and invade uterine glands (endoglandular trophoblasts), which are thereby opened towards the intervillous space from the very beginning of pregnancy onwards [2]. Hence, studying the consequences of uterine secretion products on trophoblasts cultured under flow could be another exciting field of research. Implementation of such specific culture media should be thoroughly characterized by continuous monitoring of component consumption by the cells. 

Investigation of the “trophoblast mechanosome” will be another intriguing future direction, which should include gestational age-dependent variations, fetal sex, and maternal confounders such as smoking, diabetes, and/or obesity. Characterization of the trophoblastic mechanosome and pathologic aberrations thereof could be subject to therapeutic interventions. However, development of such drugs should not only include the therapeutic potential, but also the consequences on human pregnancy. Transfer of mechanosome modulators through the placental barrier may have adverse effects on the fetoplacental circulation, leading for example to disruption of the balanced pressure gradient between the maternal and fetal circulations [7].

## Figures and Tables

**Table 1:** Overview of flow culture approaches to study trophoblast differentiation and fusion.

Shear Stress/ Flow Rate	Cells Used	Co-Cultivation	Incubation Time	Reference
30 µL/h	JEG-3	HUVECs	68 h	Lee et al. (2015) [28] **
0.001–0.12 dyn/cm² 2–5 µL/min	BeWo	-	15 min–12 h	Miura et al. (2015) [16] *
	HVTs	-		
5.2 dyn/cm²	JEG-3	HBMECs	10–21 days	McConkey et al. (2016) [27] ***
1.67 µL/min	BeWo b30	HPVECs	72 h	Blundell et al. (2016) [29] **
1 dyn/cm² 5.19 mL/min	human primary term trophoblasts	-	15 min–72 h	Lecarpentier et al. (2016) [21] *
0.001–1 dyn/cm² ≈0.5 mL/min	BeWo	-	96 h	Sanz et al. (2019) [17] *
0.1, 0.2, 0.5 mL/min	rTSCs	-	48 h	

human villous trophoblasts (HVTs), rabbit trophoblastic stem cells (rTSCs), human umbilical vein endothelial cells (HUVECs), human brain microvascular endothelial cells (HBMECs), human primary placental villous endothelial cells (HPVECs), * circulating flow loop; ** one-time inlet-to-outlet flow system; *** rotating wall vessel (RWV) bioreactor.

**Table 2:** Overview of flow culture approaches to study trophoblast motility.

Shear Stress/ Flow Rate	Cells Used	Co-Cultivation	Incubation Time	Reference
5 dyn/cm^2^	JEG-3	-	<48 h	Lanz et al. (2001) [15] *
15 or 30 dyn/cm²	macaque trophoblasts (GD 40-100); human term trophoblasts	-	24 h	Soghomonians et al. (2002) [22] *
1–30 dyn/cm²	macaque trophoblasts (GD 40-100); human term trophoblasts	UtMVECs	12 h	Soghomonians et al. (2005) [23] *
0–30 dyn/cm²	human first trimester trophoblasts	-	24 h	Liu et al. (2008) [19] *
15 dyn/cm²	macaque trophoblasts (GD 40-65)	UtMVECs	12 h	Cao et al. (2008) [24] *
15 dyn/cm²	human first trimester trophoblasts	HUVECs	12 h	Liu et al. (2009) [20] *
0.5 and 3 dyn/cm²	JAR, SGHPL-4, HUVECs	-	15 h	James et al. (2011) [18] **
0.02, 1, 2 dyn/cm²			24 h	
0.5 and 3 dyn/cm²	primary EVTs, JAR	HUVECs	31 h	
5 and 7 dyn/cm²	JAR		13 h	
0.5–6 dyn/cm²	SGHPL-4	HUVECs	7 h	James et al. (2012) [14] **

human umbilical vein endothelial cells (HUVECs), gestational day (GD), uterine microvascular endothelial cells (UtMVECs), extravillous trophoblasts (EVTs). * circulating flow loop; ** one-time inlet-to-outlet flow system;

## Abbreviations

11β-HSDs	11β-hydroxysteroid dehydrogenase enzymes
2-NBDG	2-[N-(7-nitrobenz-2-oxa-1,3-diazol-4-yl)amino]-2-deoxy-D-glucose
ANGPTL4	Angiopoietin-like 4
cAMP	Cyclic adenosine monophosphate
CREB	Activated cAMP response element binding protein
CTBs	Cytotrophoblasts
CYP1B1	Cytochrome P450 1B1
ERM	Ezrin-Radixin-Moesin
ERVFRD-1	Syncytin-2
ERVW-1	Syncytin-1
EVTs	Extravillous trophoblasts
GCM1	Transcription factor glial cell missing 1
GD	Gestational day
GLUT1	Glucose transporter 1
HBMECs	Human brain microvascular endothelial cells
hCG	Human chorionic gonadotropin
HPVECs	Human primary placental villous endothelial cells
HUVECs	Human umbilical vein endothelial cells
HVTs	Human villous trophoblasts
IVS	Intervillous space
PGF	Placental growth factor
PLIN2	Perilipin 2
PPAR	Peroxisome proliferator-activated receptor
rTSCs	Rabbit trophoblastic stem cells
RWV	Rotating wall vessel
sFlt-1	Soluble fms-like tyrosin kinase-1
STB	Syncytiotrophoblast
UtMVECs	Uterine microvascular endothelial cells
